# Phospho-ubiquitin: rewriting the ubiquitin code

**DOI:** 10.1186/s13024-026-00947-z

**Published:** 2026-05-21

**Authors:** Tauseef R. Butt

**Affiliations:** Progenra Inc, 271A Great Valley Parkway, Malvern, PA 19355 USA

## From degradation tag to signaling language

The ubiquitin system is historically viewed as a degradative pathway in which K48-linked polyubiquitin chains target proteins for proteasomal destruction. That narrow interpretation has been replaced by a far more expansive understanding. Ubiquitination is now recognized as a versatile and information-rich post-translational signaling system. It regulates chromatin dynamics, receptor trafficking, endocytosis, DNA repair, inflammation, and signal transduction.

As articulated in the expanded lexicon of the ubiquitin code, distinct ubiquitin chain topologies assembled through lysines K6, K11, K27, K29, K33, K48, and K63 encode discrete biological outcomes. Chain length, linkage type, and branching architecture together form a combinatorial signaling language [1]. A transformative addition to this language was the discovery that ubiquitin itself can be phosphorylated at Ser65 by PTEN-induced kinase 1 (PINK1) [2]. This single modification introduced an entirely new regulatory dimension: the phospho-ubiquitome. Unlike canonical ubiquitin signals, Ser65 phosphorylation converts ubiquitin from a modifier into an enzymatic activator, fundamentally redefining its functional repertoire. Phosphorylation does not merely decorate the ubiquitin code; it rewrites its grammar.

## PINK1–parkin signaling: a feed-forward alarm system

PINK1 functions as a sensor of mitochondrial integrity. Under basal conditions, PINK1 is imported into healthy mitochondria and rapidly degraded. Loss of mitochondrial membrane potential stabilizes PINK1 on the outer mitochondrial membrane (OMM), where it phosphorylates pre-existing ubiquitin at Ser65 [2]. Phosphorylated ubiquitin (p-Ub) acts as a high-affinity ligand for Parkin, an RBR (ring between ring) E3 ubiquitin ligase maintained in an autoinhibited conformation by its N-terminal ubiquitin-like (Ubl) domain. PINK1 activates Parkin through dual phosphorylation. It phosphorylates ubiquitin at Ser65. PINK1 also phosphorylates Ser65 within Parkin’s own Ubl domain. Binding of p-Ub to Parkin’s RING1 domain induces conformational rearrangements that release autoinhibition and activate the catalytic RING2 domain. Structural studies have elucidated the feed-forward architecture underlying this activation mechanism [3]. In a pharmacological sense, p-Ub acts as ligand, and Parkin as a receptor. The result is a powerful amplification loop. Activated Parkin ubiquitinates numerous OMM substrates, assembling predominantly K63-linked chains, along with K11, K6, K48, and branched linkages [4]. Within this architecture, phospho-ubiquitin serves two mechanistically distinct roles:

1. **Allosteric activator** of Parkin.
2. **Chain building block** incorporated into growing p-poly-ubiquitin assemblies.

**Fig. 1:**
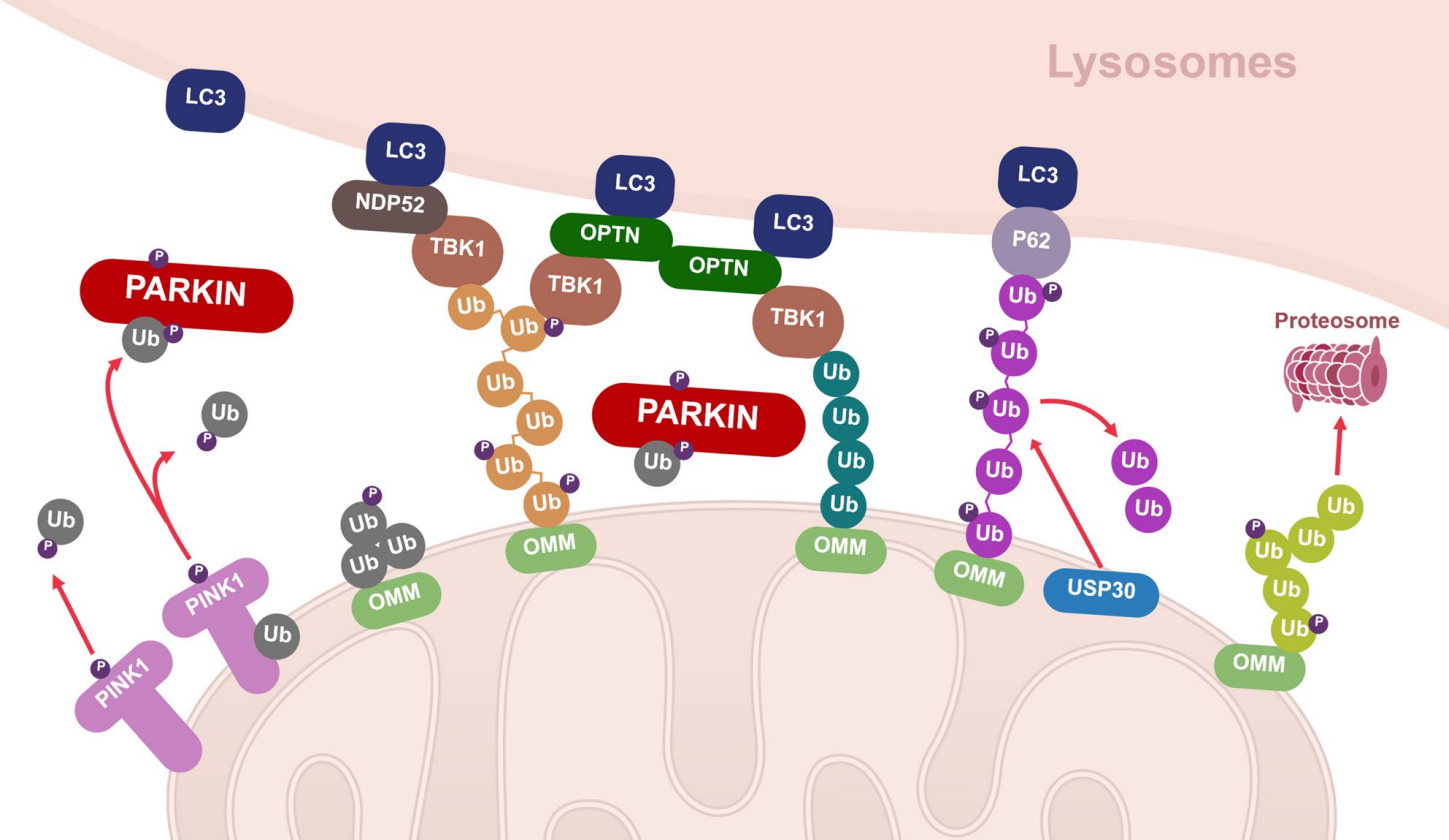
Multiple mono-ubiquitins or p-poly-ubiquitin chains synthesized on mitochondria to initiate mitophagy. PINK1 phosphorylate neighboring ubiquitin attached to mitochondrial outer membrane (OMM) proteins. Data from different labs show that Parkin/PINK1 system synthesizes and phosphorylates different types of poly-ubiquitin chains [5, 6]. Diseased mitochondria proteins may contain mono-ubiquitin, multi-mono-ubiquitin. The ubiquitin chains are recognized by autophagy receptors, such as OPTN, NDP52 and P62, whose functions are further modulated by TBK1. Conversely, the mitochondrial DUB USP30 acts as a negative regulator of mitophagy. Different colors in poly-ubiquitin chains signifies linkage specific poly-ubiquitin designed on OMM proteins. Presence of K6, K11, K48, K63 homotypic linkages as well as K6/K48 and K48/K63 branched ubiquitin linkages are well established (see ref [3, 9, 10])

This dual functionality distinguishes p-Ub from canonical ubiquitin modifications. It is not merely a degradation tag; it is a catalytic signal that amplifies mitochondrial dysfunction and mediates mitophagy (see Fig. 1).

## Beyond parkin: is phospho-ubiquitin a general signal?

Parkin is the best-characterized member of the RBR E3 ligase family, which comprises approximately 18 ligases in humans. Yet the conceptual implications of phospho-ubiquitin extend beyond a single enzyme.

Key unresolved questions include:

- Does p-Ub regulate additional RBR ligases?
- Are there tissue-specific p-Ub and p-poly-ubiquitin signatures?
- Does p-Ub function outside mitophagy, for example in transcriptional control or inflammatory signaling?
- Does p-Ub alter chain topology preferences for DUBs?

What is the fundamental difference between native and phospho-poly-ubiquitin chains? Emerging data suggests that other ligases can incorporate p-Ub into ubiquitin chains and that PINK1 may phosphorylate additional substrates within the pathway [7]. However, whether p-Ub functions as a broadly generalizable allosteric signal remains an open question. The observation that p-Ub chains are resistant to many of the DUBs tested provides proof that nature designed p-Ub system to ensure that damaged mitochondria are removed by mitophagy [8]. Sealing the fate of p-poly-ubiquitinated proteins is one way nature protects the target protein from ~ 100 DUBs present in cells. If confirmed, phospho-ubiquitin would represent not merely a mitochondrial alarm bell, but a new signaling currency.

## Phospho-ubiquitin in neurodegeneration

Neurons are particularly dependent on mitochondrial function. Their high energetic demand, reliance on oxidative phosphorylation, and post-mitotic state render them especially vulnerable to mitochondrial dysfunction. Age-associated decline in mitophagy leads to accumulation of damaged mitochondria, increased reactive oxygen species, and impaired bioenergetics [4].

Autosomal recessive mutations in PINK1 or Parkin cause early-onset Parkinson’s disease, directly linking p-Ub signaling to neuronal survival. Dopaminergic neurons of the substantia nigra are particularly susceptible due to their metabolic burden and dopamine-associated oxidative stress. Defective PINK1–Parkin signaling has also been implicated across major neurodegenerative disorders, including Parkinson’s disease (PD) and Alzheimer’s disease (AD). Increase p-Ub is associated with neuronal Lewy body-type α synuclein pathology, but not with glial cytoplasmic inclusions of α synuclein as seen in multiple system atrophy [9].

Importantly, p-Ub also intersects with mitobiogenesis. PGC-1α is a master transcriptional coactivator driving mitochondrial gene expression. PARIS, a transcriptional repressor of PGC-1α, is degraded through PINK1/Parkin signaling. PINK1 phosphorylates PARIS, promoting Parkin-mediated ubiquitination and proteasomal clearance [10]. Recent biochemical and genetic data (unpublished) from different labs demonstrate that PARIS plays a critical role in mitobiogenesis. Thus, the pathway achieves an elegant symmetry: damaged mitochondria are removed, and new mitochondria are generated. Mitophagy and mitobiogenesis are coupled by p-Ub signaling.

## The biomarker paradox

Current biomarkers for neurodegeneration, α-synuclein, tau, and amyloid-β, typically detect aggregated proteins after substantial neuronal loss [11]. In contrast, phospho-ubiquitin reflects upstream mitochondrial dysfunction, potentially decades before clinical manifestation.

Analysis of large cohort of AD and PD patient sample by p-Ub antibody and Mesoscale discovery platform did not demonstrate sufficient discrimination between level of p-Ub in diseased vs. control plasma samples. However, analysis CSF from PD cohort showed better discrimination between disease and control samples [12]. There is no doubt that p-Ub is a marker for mitochondrial dysfunction. The question remains whether the antibody is selective to capture all forms of p-polyubiquitinated proteins or Mesoscale platform is sensitive enough to detect p-Ub as a biomarker.

We believe that p-Ub remains a promising biomarker, however, technical challenges complicate interpretation:

- P-poly ubiquitin chains exhibit mixed and branched architectures.
- Ser65 phosphorylation epitope can be sterically masked in K63-linked p-poly-ubiquitin chains, thus reducing the p-ploy-K63 chain signal.
- Commercial antibodies poorly recognize K63-linked p-poly-ubiquitin.
- Many proteomic datasets rely on artificial mitochondrial depolarization models rather than physiological tissue.

Thus, the biomarker signal is biologically compelling but technically challenging.

## Structural complexity of the phospho-ubiquitome

Ubiquitin mass spec proteomics following induction of mitophagy is accompanied by dramatic increases in K63- and K11-linked ubiquitin chains, 70 and 14-fold respectively in primary cortical mouse neurons [5]. These assemblies frequently contain mixed linkages and partial phosphorylation, generating structurally heterogeneous polymers (see Fig. 1).

The phospho-ubiquitome is therefore multidimensional:

- Linkage type.
- Chain length.
- Branching topology.
- Stoichiometry of phosphorylation.

Each variable alters signaling output. Phosphorylation layered onto complex chain architectures creates a combinatorial signaling matrix whose endogenous structure in aging human tissue remains largely unresolved. Mono-p-Ub has a unique function, to jumpstart and feed forward the Parkin poly-ubiquitin chain formation. P-Ub will become a true biomarker when tools are developed to isolate p-poly-ubiquitinated α synuclein, tau, TDP43 and huntingtin. The challenge ahead is analytical rather than conceptual: we must define the architecture of phospho-polyubiquitin chains in physiological systems, not just in chemically depolarized cell lines.

## Therapeutic convergence

Small molecules that activate Parkin through molecular glue mechanisms have been described [13]. Pharmacological activation of PINK1 ameliorates pathology in preclinical Parkinson’s disease models (Mitokinin/AbbVie). Because p-Ub levels directly reflect PINK1–Parkin activity, they serve as a pharmacodynamic biomarker for:

- Early detection of disease and of mitochondrial dysfunction.
- Patient stratification for pathway-targeted therapies.
- Monitoring target engagement in clinical trials.

Realizing this potential requires next-generation assays capable of discriminating mono-phospho-ubiquitin from structurally complex p-poly-ubiquitin chains in blood, urine, and cerebrospinal fluid. Applications of affinity matrices that selectively isolate and enrich p-poly-ubiquitinated pathogenic proteins will open development of novel therapeutics and biomarker field [14].

## A conceptual shift

The PINK1–Parkin pathway exemplifies a profound biological transition. A molecule once considered a passive degradation tag becomes an active signal that senses mitochondrial damage, amplifies physiological responses, and coordinates organelle turnover with biogenesis. Ubiquitin phosphorylation is not an incremental extension of the ubiquitin code. It is a rewriting of its grammar. The remaining challenge is to chart the endogenous p-Ub landscape across tissues, aging, and disease. Only then will we determine whether p-Ub functions solely as a mitophagy trigger or as a broader signaling hub in human physiology. The paradigm has shifted. Now the tools must catch up.

## Data Availability

The perspective reviews the data generated by other researchers as well in authors company/lab to draw conclusions and proposals made in the perspective.

## Abbreviations

p-Ub: Phospho-ubiquitin
PD: Parkinson’s disease
AD: Alzheimer’s disease
p-poly-ubiquitin: Phospho-poly-ubiquitin
Ubl: Ubiquitin-like
